# Development of vaccine formulations: past, present, and future

**DOI:** 10.1007/s13346-021-00924-7

**Published:** 2021-02-17

**Authors:** Carmine D’Amico, Flavia Fontana, Ruoyu Cheng, Hélder A. Santos

**Affiliations:** 1 Drug Research Program, Division of Pharmaceutical Chemistry and Technology, Faculty of Pharmacy, University of Helsinki, FI-00014 Helsinki, Finland; 2 Helsinki Institute of Life Science (HiLIFE), University of Helsinki, FI-00014 Helsinki, Finland

**Keywords:** Vaccination, Immunotherapy, Nanoparticles, Microneedles, Drug delivery

## Abstract

The current situation, heavily influenced by the ongoing pandemic, puts vaccines back into the spotlight. However, the conventional and traditional vaccines present disadvantages, particularly related to immunogenicity, stability, and storage of the final product. Often, such products require the maintenance of a “cold chain,” impacting the costs, the availability, and the distribution of vaccines. Here, after a recall of the mode of action of vaccines and the types of vaccines currently available, we analyze the past, present, and future of vaccine formulation. The past focuses on conventional formulations, the present discusses the use of nanoparticles for vaccine delivery and as adjuvants, while the future presents microneedle patches as alternative formulation and administration route. Finally, we compare the advantages and disadvantages of injectable solutions, nanovaccines, and microneedles in terms of efficacy, stability, and patient-friendly design.

**Graphical abstract:**

Different approaches to vaccine formulation development, the conventional vaccine formulations from the past, the current development of lipid nanoparticles as vaccines, and the near future microneedles formulations are discussed in this review.

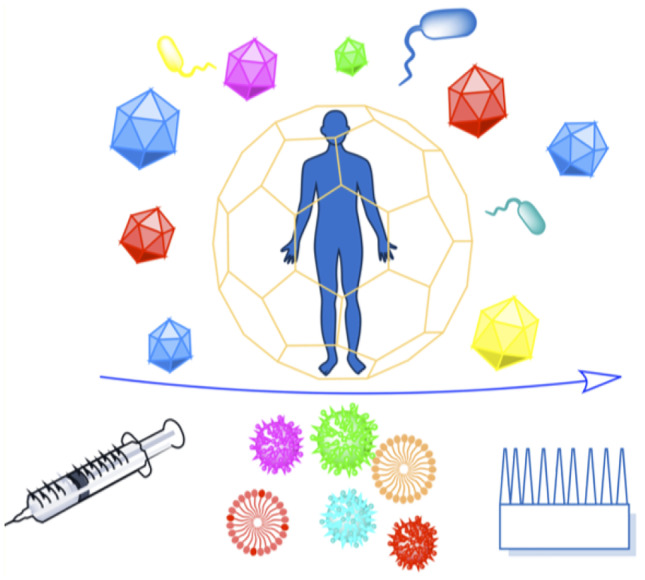

## Introduction

The challenging months of 2020 have brought to the forefront the critical issues associated with the discovery and formulation of effective treatments and, ultimately, a vaccine during epidemics and pandemics [1]. The emergence of a new type of respiratory coronavirus (severe acute respiratory syndrome, SARS, CoV, or 2019 novel CoV) and its growth in the present-day pandemic recall the previous two experiences with CoV, namely SARS-CoV and Middle East Respiratory Syndrome Coronavirus (MERS-CoV) [1, 2]. However, the development of effective vaccines for SARS-CoV or MERS-CoV was slowed or abandoned once the epidemic was controlled [3, 4]. The critical analyses on pandemic preparedness after the H1N1 pandemic highlighting the failure to distribute enough vaccines where they were needed, when they were needed, had not been implemented before the emergence of SARS-CoV2 [5]. This has caused a delay in the discovery and formulation of candidate vaccines for SARS-CoV2, requiring an unprecedented effort by public (academia and government bodies) and private (industrial) to fast track the development of vaccines [6].

The current pandemic highlighted also the challenges related to timely distribution of vaccines for seasonal flu or other diseases, together with the problematic “cold chain” [7]. These challenges are heavily dependent on the vaccine formulations and their features (Table 1), and thereby, on pharmaceutical technology research and innovations.

**Table 1:** Comparison between the characteristics of the different vaccine formulations

Features	Injections	Microneedles	Nanovaccines	Oral solutions
Mode of administration	Needle	(Micro)needle	Injection/inhalation	Liquid oral solution
Onset	Fast	Fast	Fast	Slow
Self-administration	No	Yes	No	Yes
Storage conditions	Cold chain	Room temperature	Formulation-dependent	Cold chain
Pain	Painful	Painless	Painful/Painless	Painless
Patient compliance	Poor	Good	Medium	Very good
Safety	Poor	Good	Good	Good
Usability	Moderate	Good	Moderate/Good	Good

In this review, we will first introduce the immunological mechanisms at the base of vaccination, followed by a discussion on the classes of vaccines available. The main body of the article will analyze the past conventional formulations, the present solutions entertained by nanotechnology, and the future developments.

### Immunological context of vaccination

**Fig. 1:**
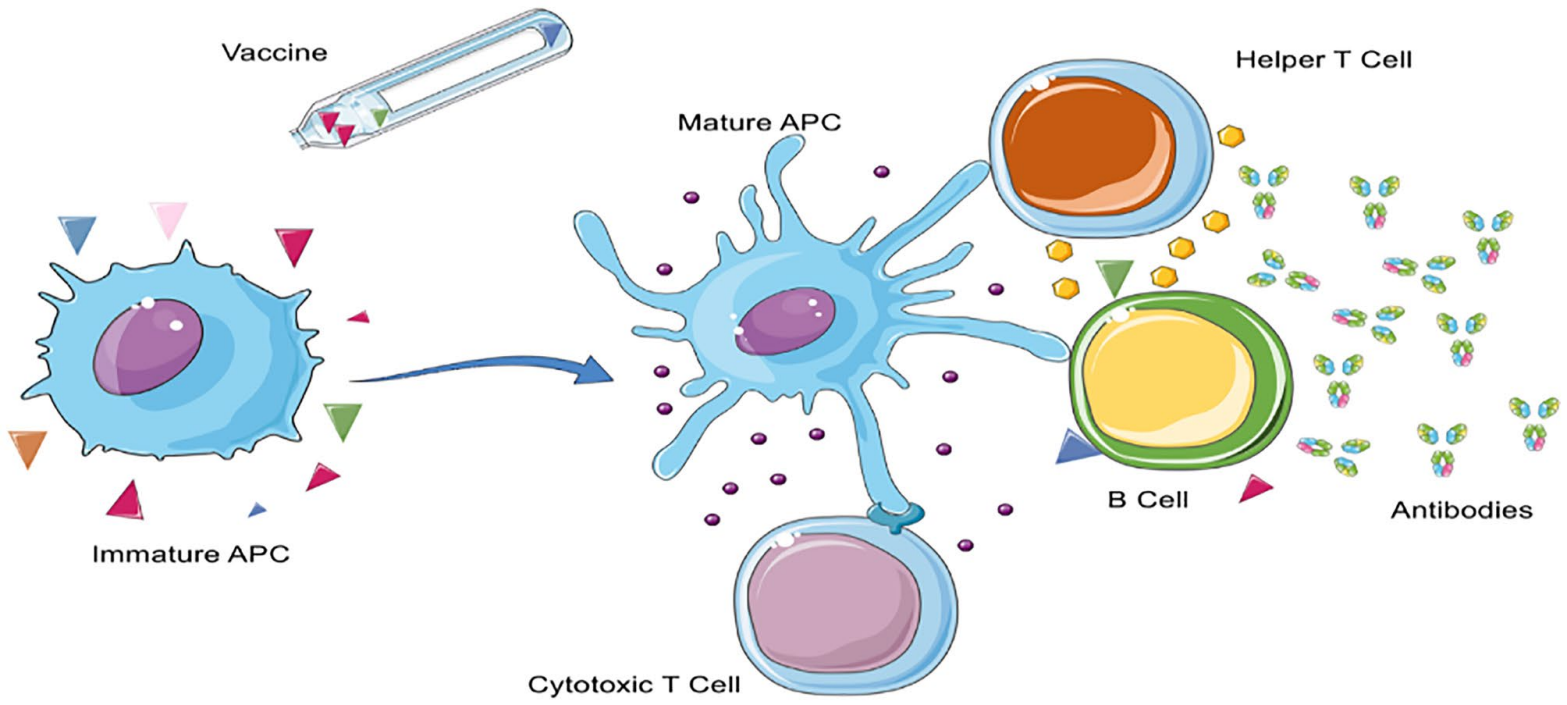
Immune activation after vaccination. The antigens and adjuvants contained in the vaccine formulation are taken-up by immature APC or B cell. The APC processes the signal and achieves a mature phenotype, further transmitting the signal to cytotoxic T cells, helper T cells, and B cells. The endpoint in a successful vaccination is the development of antigen-specific antibodies and cytotoxic T cells. Figure created from elements of Servier Medical Art, licensed under a Creative Commons Attribution 3.0 Unported License

The paradigm of vaccination is the creation of a long-term immunization against one or more antigens specific for a pathogen or cancer cell through the development of antibodies and cytotoxic T cells. The process can be summarized into 3 steps: (1) uptake of antigens and adjuvants from antigen presenting cells (APCs), (2) maturation of the APCs, and (3) priming of antigen-specific B and T cells with the production of antibodies and cytotoxic T cells (Fig. 1) [8].

**Fig. 2:**
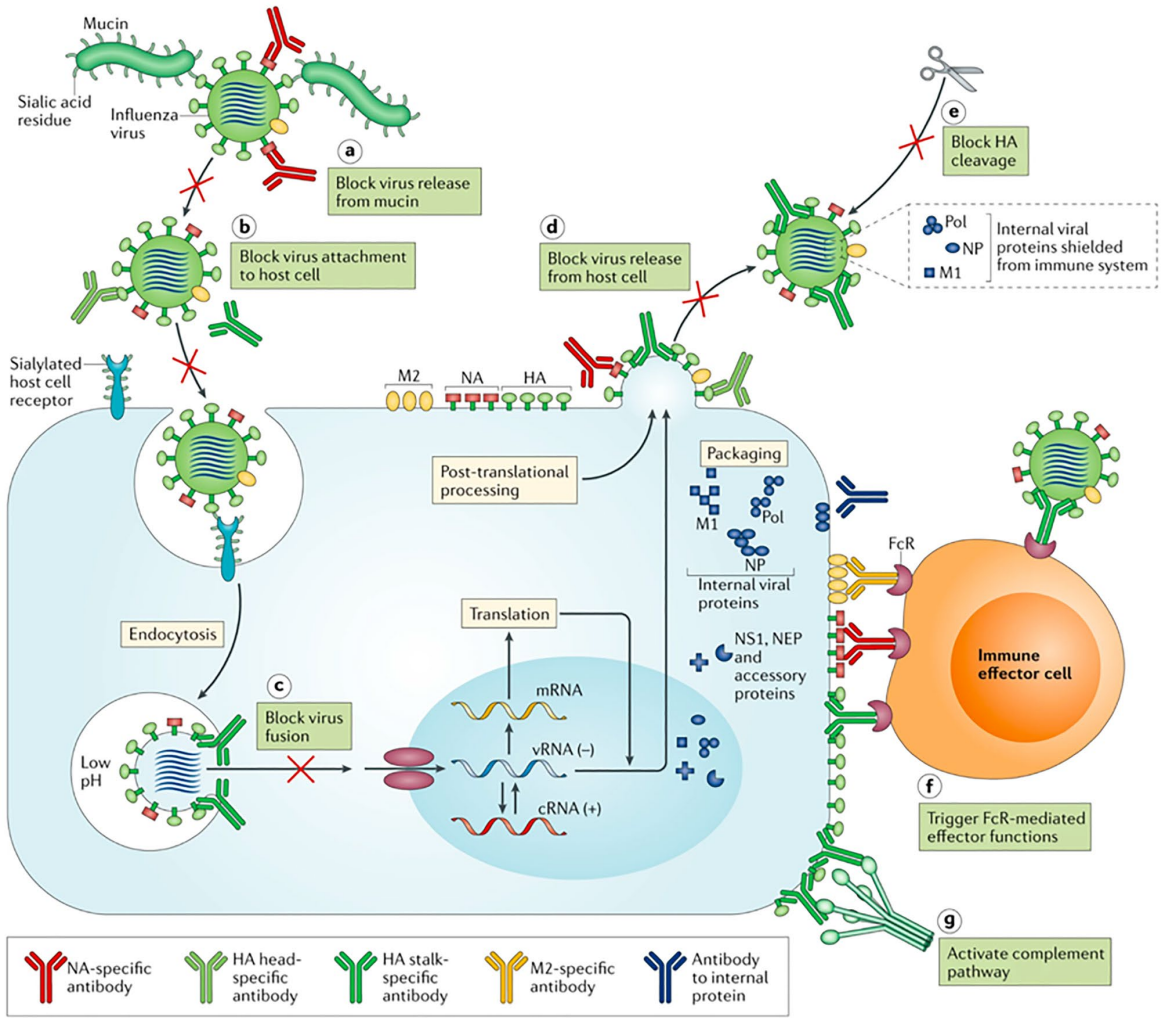
Mode of action of antibodies against viruses. The seven different mechanisms by which antibodies can block a viral infection (the examples in the figure refers to flu). Reproduced with permission from ref. [14]

APCs are immature dendritic cells, macrophages, B cells, or even immune fibroblasts, which can take-up antigens and are activated by endogenous or exogenous danger signals [9–11]. Upon activation, the APCs, particularly dendritic cells, assume a mature phenotype while processing the antigens into peptides suitable for the expression on major histocompatibility molecules (MHCs) I or II. At the same time, the APC is presenting co-stimulatory signals (e.g., CD80 or 86) and secreting proinflammatory cytokines [12]. Finally, naïve T cells interact with MHC and are primed into cytotoxic or helper T cells. B cells get activated upon interaction of their B cell receptor (BCR) with soluble or bound antigens, then leading to the differentiation into plasma or memory B cells and the production of antigen-specific antibodies [13]. Furthermore, the activation process can be dependent on the presence of helper T cells or independently for any other signal. Antibodies can fight a viral infection by attaching on the surface of the virus, creating steric hindrance, preventing viral infection in the cells, preventing the virus release from the infected cells, blocking the cleavage of hemagglutinin, activating complement, and flagging the virus to phagocytes for the elimination (Fig. 2) [14].

As for cytotoxic T cells, their role is to recognize and kill virus-infected or cancer cells, as well as to release interferon-γ and tumor necrosis factor-α [15, 16]. The type of vaccine, antigen, adjuvant, and route of administration chosen all have an effect on the type and magnitude of the immune response primed, as well as on the duration of the immunological memory.

### Types of vaccines

**Fig. 3:**
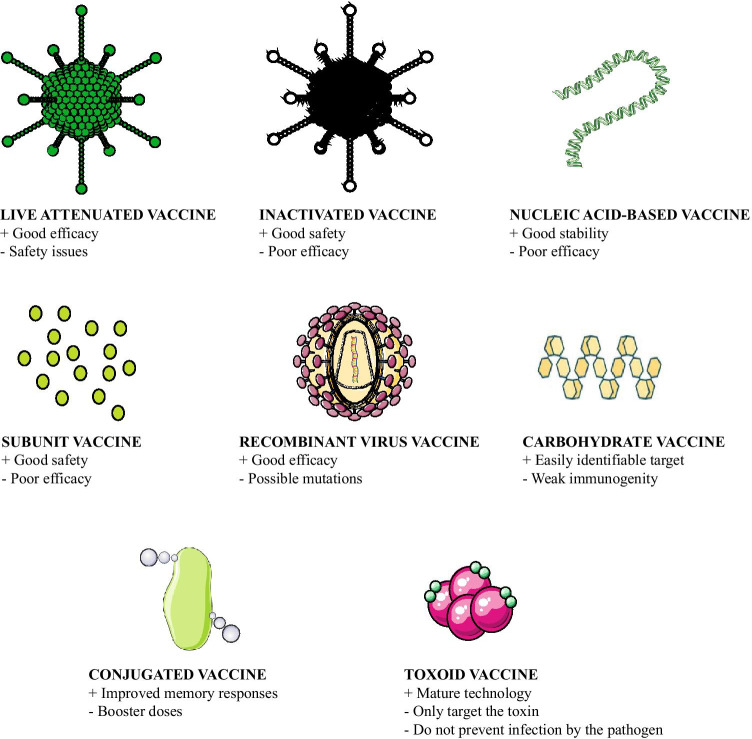
Classes of vaccines, their main advantages, and disadvantages. Figure created from elements of Servier Medical Art, licensed under a Creative Commons Attribution 3.0 Unported License

Currently there are eight different classes of vaccines, distinct in origin, composition, and immunogenicity (Fig. 3).

Live-attenuated vaccines are the first type of vaccines developed, and they have successfully eradicated smallpox, almost eradicated poliomyelitis, and contribute to control the worldwide cases of measles while being less effective for chronic infections [17]. Furthermore, this class of vaccines is associated with risks connected with the safety and efficacy of the vaccine. Live-attenuated vaccines either are usually closely related viruses that are not pathogenic for human, or less severe strains, or are obtained after repeated culturing in cells from a species not permissive for the virus [17]. Importantly, these vaccines are the only class not requiring any additional adjuvant to be co-administered. The safety concerns are related to possible reversion to virulent wild type [18]. Furthermore, these viruses are sensitive to storage conditions and demand a well-kept cold chain [19]. Inactivated or “killed” vaccines are prepared by inactivating the pathogen through heat, radiation, or use of chemical compounds (e.g., formalin) [20]. Inactivating means to destroy the ability of the virus to replicate in the human body while keeping all the antigens present in the viral structure [20]. A variety in the immune response according to the source of the antigen, the need for adjuvants, and the lower efficacy are the downsides of this class of vaccines.

The third class of vaccines in order of discovery is toxoids [21]. Toxoid vaccines are prepared from the toxins secreted from bacteria (e.g., tetanus and diphtheria). It is a mature technology, and vaccines for tetanus and diphtheria are commonly administered worldwide. They are not highly immunogenic, and they require the formulation with an adjuvant, as well as multiple administrations. However, they show an excellent stability profile [22]. Starting from 1970, the use of modern technologies in microbiology allowed for the isolation of carbohydrate-based vaccines, which are mainly targeting the bacterial capsule polysaccharides, but also viruses and cancer, and are nowadays produced from synthetic methods to obtain homogenous glycans [23]. In order to increase their immunogenicity, particularly in children, they are often conjugated to a carrier protein [23]. Conjugated vaccines are formed by the conjugation of a protein to a carbohydrate to increase its immunogenicity and stability [24]. Often, they are classified together with the carbohydrate antigens. They can be conjugated to toxoids from tetanus.

Recombinant vector vaccines are produced by inserting the sequence coding for the desired antigen from a pathogen inside another vector as foreign transgene [25]. This type of vaccine shows good immunogenicity. However, the presence of a foreign transgene in the vector genome may lead to evolutionary mutations that decrease the immunogenicity and thereby the efficacy of the vaccine [25]. Subunit vaccines are composed by protein immunogens, or antigens, usually produced through recombinant technologies [26]. They show an excellent safety profile and can be modified to change properties facilitating the development of stable formulations. However, the downside is their limited immunogenicity when compared with live-attenuated vaccines. Thereby, they often require the formulation together with adjuvants, to increase their efficacy [26].

Finally, nucleic acid–based vaccines have been developed in the past 30 years, with strong hype on their good stability and fast production, safety profile compared with live-attenuated viruses, not inducing neutralizing antibodies, and ensuring that the immune response is directed against the transgene product and not partially against the viral coating [27, 28]. mRNA-based vaccines are currently approved as vaccines for SARS-CoV2 (Moderna, Pfizer/BioNTech) [29].

A considerate analysis of formulation from a pharmaceutical technology perspective is deemed essential to provide the best characteristics to the vaccine in terms of efficacy, but also manufacturing, storing, and distribution [30]. In this review, we are presenting three different approaches to vaccine formulation development, the conventional vaccine formulations from the past, the current development of lipid nanoparticles as vaccines (e.g., Moderna and CureVac), and the near-future microneedle formulations.

## Past: conventional vaccine formulations

Conventional vaccine formulations have shaped the world, contributing to the eradication of small pox, and to control other infective diseases (e.g., measles, mumps, tetanus). Most of us have been vaccinated at some point in life; usually, we routinely get tetanus vaccine rechallenge, and we get a yearly vaccination to protect us from the new strains of flu. The conventional vaccine formulations are effective mainly against pathogens which conserve their antigenic profile (e.g., measles and mumps)[21]. A lower efficacy and the need to widen the antigenic content of the vaccine or administer new vaccines every year are found for highly mutant pathogens (e.g., influenza virus and pneumococcus). Finally, the major concern about conventional vaccines is their inefficacy toward pathogens that change their antigens after their infection (HIV). Furthermore, conventional vaccines and the adjuvants used in the formulations are mainly directed to elicit a humoral, B cell-mediated, immune response. However, in multiple cases, either B cells alone are not enough to handle the pathogen and need support from helper T cells or the pathogen infection and residence site are intracellular, requiring cytotoxic T cells for its elimination [21]. Importantly, current vaccines, particularly the ones administered to elderly population, are gender-biased; the efficacy of such vaccinations (flu and pneumococcus to begin with) is higher in women than men, a worrying factor, considering that SARS-CoV2 is more severely affecting men [31, 32]. The current situation, with the ongoing pandemic of SARS-CoV2, has highlighted once again the importance of having an effective vaccination to immunize the general population worldwide.

Conventionally administered vaccines are mostly administered through injection, intramuscular, subcutaneous, or intradermal [33]. Thereby, they usually require specific conditions for the transport and storage [33]. The formulation process is thereby based on the preparation of solid powder to be reconstituted before use. The two main focuses in the formulation development for vaccines are the stability of the final product, which will influence the storage conditions, and the addition of adjuvants, of particular interest in subunit, carbohydrate, or toxoid-based vaccines to increase their immunogenicity.

Despite the successes achieved in controlling smallpox and other diseases, there are some limitations associated with conventional vaccines, demonstrating the need for better and safer vaccines.

### Adjuvants for preparation of vaccines

As for the adjuvants, regulatory authorities have specific guidelines regulating the selection and quality control of the adjuvant, including assays to evaluate the stability of the adjuvant alone and of the adjuvant-antigen complex [34]. Furthermore, the adjuvant alone and the final formulation should go through extensive preclinical and clinical assessment of safety and efficacy, measured in the ability to stimulate the immune system against the pathogen [34]. The list of adjuvants approved from the regulatory agencies includes 9 adjuvants [35]. The most used adjuvant is alum, made of particulate aluminum salts, which form a depot in the site of administration. The exact mode of action of alum is still investigated and includes multiple pathways [35]: the presentation of the antigen on an alum particle improves the interaction with APCs; alum itself can interact with lipids on APCs membrane, activating the cells, and alum activates inflammasome, resulting into activation of innate immunity. Alternatively, oil-in-water emulsions (e.g., MF59) do not directly interact with APCs but modify the immune contexture surrounding the administration spot facilitating the migration of APCs [36]. The size of the emulsion droplet is of the utmost importance for the efficacy of the vaccination; too small particles have lower efficacy than 160 nm droplets [36]. Monophosphoryl lipid A (MPLA) derives from bacteria and activates toll-like receptor 4. MPLA is nowadays produced as synthetic analogue by synthesis instead of relying on the extraction from bacteria [35]. Saponins are natural molecules extracted from plants and are usually combined with cholesterol to lower their toxicity. They increase the uptake of the antigen by APCs by interacting with cholesterol-rich areas on the cell membrane of APCs. Finally, virosomes, used for flu vaccines, are made of liposomes with structure resembling a virus, due to their formulation from empty virus particles (usually from influenza virus) [35]. Other types of adjuvants are currently in clinical development, including DNA sequences as CpG, polyelectrolytes, and outer membrane vesicles derived from bacteria [35].

### Stability aspects of vaccines

**Fig. 4:**
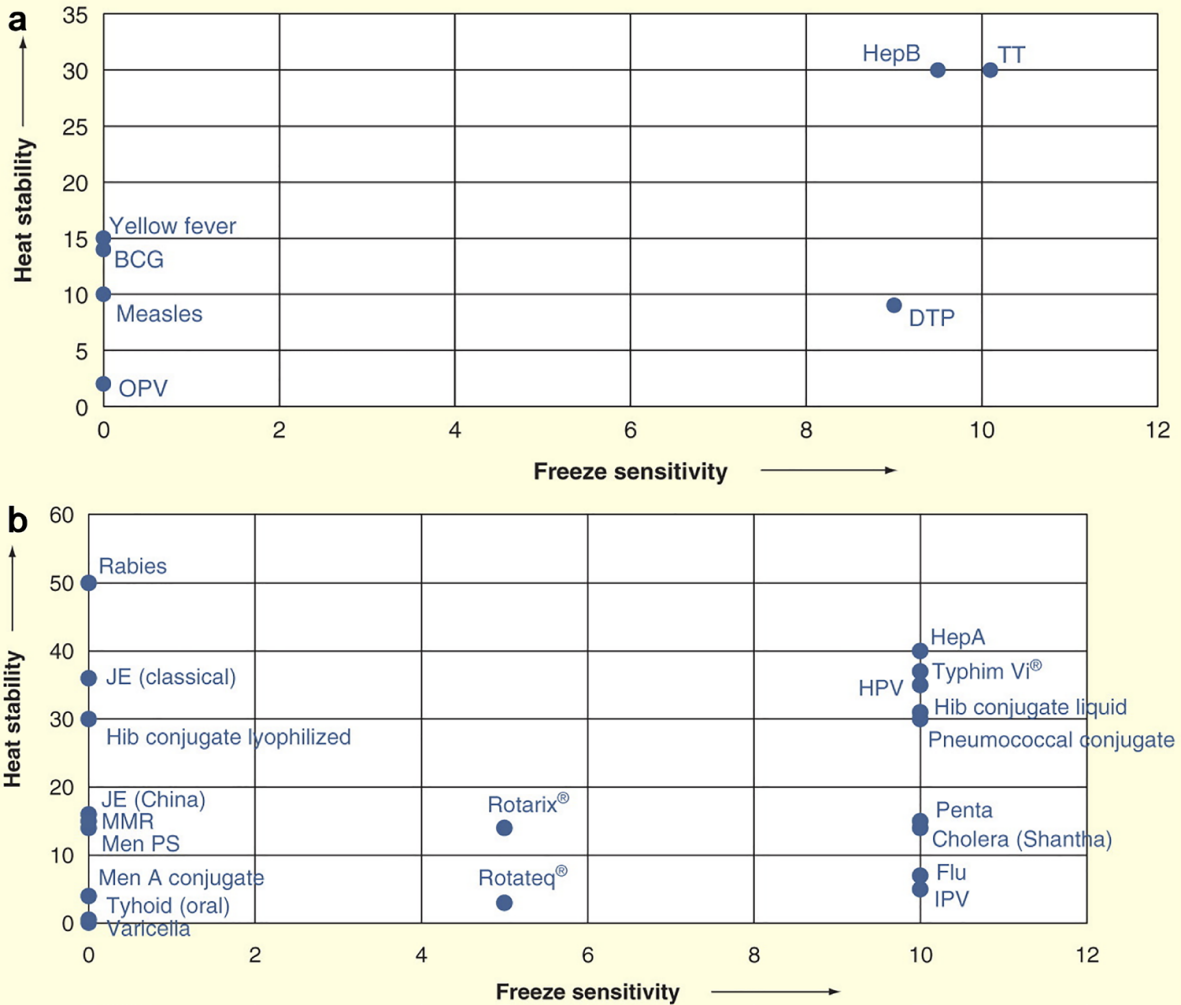
Stability of common vaccines to freeze and heat stress in ordinal scale. **a** Conventional vaccines. **b** Vaccines newly introduced in clinical practice. BCG Bacille Carmen Guerin, DTP diphtheria–tetanus–pertussis, Hep A hepatitis A, HepB hepatitis B, OPV oral poliomyelitis vaccine, Hib: Haemophilus influenza type b, HPV human papilloma virus, IPV inactivated polio vaccine, JE Japanese encephalitis, Men A meningitis A, Men PS meningitis polysaccharide, MMR measles–mumps–rubella, Penta DTP + HepB + Hib, Rotarix® and Rotateq® rotavirus vaccine, TT tetanus toxoid, Typhim Vi® typhoid polysaccharide vaccine Reproduced from ref. [38], under the terms of the Creative Commons Attribution License (http://creativecommons.org/licenses/by/3.0)

The main disadvantage of conventional vaccines, the storage requirements including cold chain, impacts both the worldwide distribution and the stockpiling [37]. Different vaccine formulations exhibit different stabilities to temperature, as summarized in ordinal scale in Fig. 4.

In particular, liquid formulations, with or without adjuvants, are more sensitive to damage from freezing; thereby, they need a carefully controlled refrigeration. On the contrary, the type of vaccine (inactivated, subunit, and toxoids) has an impact on the stability to heat. Inactivated viruses are the most sensitive to heat, while toxoids and subunits the least. This is of utmost importance for flu vaccines, and research has focused on stabilization by drying the solution into solid powder, keeping the efficacy. The techniques for the drying are spray drying, freeze-drying, spray freeze-drying, vacuum, or air-drying [37]. The obtained powders are then suitable also for alternative administration routes, including dermal, pulmonary, nasal, oral, and preventing the needle phobia [37].

The main issue arising when drying a solution containing the antigens is the sensitivity of the antigen to heat, cold, shear, and dehydration. Thereby, selected protective excipients, such as l-leucine, lactose/trehalose, and mannitol/dextran, are routinely employed in the formulative process. In particular, in the case of a type 5 human Adenovirus vector, the combination of excipients preserving the best activity after spray drying was mannitol/dextran, allowing for storage at room temperature, instead of −80°C [39]. The choice of a suitable stabilizer depends on the type of vaccine: in two types of recombinant vectors, enveloped virus or non-enveloped one, the type of stabilizer giving the best effect differed. In presence of a lipidic membrane, in an enveloped virus, trehalose resulted in higher protection of the virus, due to the formation of stronger hydrogen bonds with the lipids of the viral membrane. Conversely, for non-enveloped adenovirus, mannitol, due to its smaller size, was more effective in replacing the water molecules surrounding the capsid [40]. Thereby, a careful optimization of the formulation parameters in the first stages of the vaccine product development should include also the analysis of the best excipients to stabilize the antigens in a vitrified status. However, the formulation of antigens and adjuvants in nanoparticles or microneedles can contribute to solve the stability problem, as discussed below.

## Present: nanotechnology in vaccine development

With the development of nanotechnology, more researchers focus their interests in developing nanomaterials as promising vaccination methods, not only because nanomaterials have controlled properties, such as diameter, zeta-potential, surface morphology, and antigens loading efficiency, but also due to various nanomaterials triggered immune responses by targeted delivery in vivo [41, 42]. Different from the conventional vaccines for influenza, chickenpox, measles, mumps, and rubella that usually contain the inactivated pathogens, nanomaterial vaccines (nanovaccines) are mainly involved in subunit vaccines, containing only the necessary antigens that trigger the targeted immune response [43]. As mentioned above, subunit vaccines are safer but less immunogenic than the conventional vaccines, due to the lack of the pathogen-associated molecular patterns (PAMPs); thus, additional adjuvant or/and nanomaterial delivery systems are required for the full function of the subunit vaccines [44]. An important advantage of the nanomaterials is the appropriate protection of the antigens within the nanomaterials until they reach the targeted area where they are delivered to the antigen-presenting cells (APCs) [45]. In other words, the appropriate protection of antigens in the nanomaterials can decrease the unnecessary immune response caused by the exposition of antigens on systemic circulation [46]. Moreover, some of the nanomaterials itself can exhibit certain immunogenicity when they are internalized by the APCs [47].

Several nanomaterial delivery systems have been developed as nanovaccines, such as polymer-based nanoparticles, metallic nanoparticles, liposomes, inorganic nanoparticles, and composited nanoparticles [48–52]. Interestingly, not only the composition of these nanovaccines, such as the loading adjuvant or antigens, affect the immunogenicity, but also the properties of these nanovaccines like the diameter, shape, and surface coating have an impact on the immune response [53]. In this section, the interaction between nanovaccines’ properties and immunogenicity, the application of these nanovaccines, and their advantages and disadvantages are discussed focusing on the continuous development of such systems.

### Nanovaccine properties and their effects on immunogenicity

Since the size is one of the most important properties in nanomaterials, researchers have systematically investigated the interaction between the nanovaccines’ diameter and the immunogenicity [54, 55]. So far, there are several studies showing that nanovaccines with small diameter can be more effectively internalized by the APCs than the big ones, because the smaller diameter makes them easily transported through the epithelia and other biological barriers [56]. For example, Gutierro et al. prepared bovine serum albumin (BSA)-loaded poly(d,l-lactic-co-glycolic) acid (PLGA) nanovaccines with different diameters (200, 500, and 1000 nm). These nanovaccines were administrated (intranasally, orally, or subcutaneously) into BALB/c mice. Compared with the free antigen (1 µg, subcutaneous administration), the particle groups exhibited slightly higher serum total IgG and IgG2a/IgG1 ratio. However, the decreasing diameter did not result in increasing immunogenicity of PLGA nanovaccines. In addition, the 1000-nm nanovaccines elicited higher serum IgG antibody levels and similar IgG2a/IgG1 ratios compared with the smaller ones, which can be due to the enhanced access to the APCs and a similar antigen-presentation mechanism. Generally, the particle diameters ranging from 20 to 50 nm tend to drain to the lymphatic vessels and accumulate in the lymph nodes [57]. In addition, Li et al. administrated BALB/c mice with various zein particles distinguished from different diameter (241.4 to 879.2 nm), doses (200, 600, 800 µg), and administration route (intramuscular and subcutaneous). Interestingly, they found that the particle diameter did not have any influence on the immunogenicity after three intramuscular injections. The immune response generated was long lasting and highly specific. The repeated administration induced rapid and strong systemic recall immune responses via both intramuscular and subcutaneous routes. The immunogenicity of zein nanoparticles can be of concern when they are applied as drug delivery systems, but this also can be an advantage when they are used as nanovaccines [58]. In general, the interactions between various particle diameters and the immunogenicity are complicated dynamic processes that can also be influenced by the administration routes, types of particles, and injection doses.

**Fig. 5:**
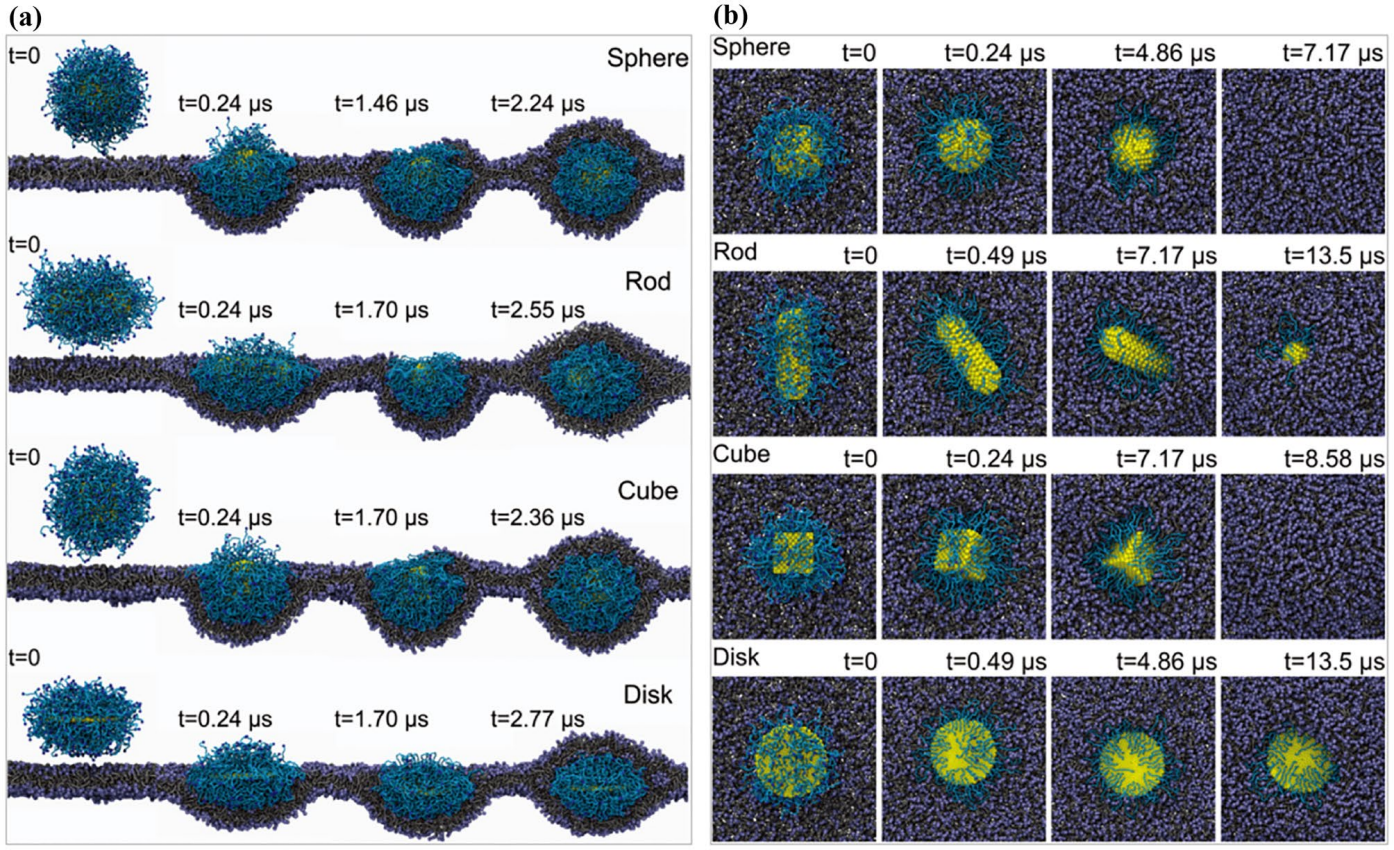
Internalization pathway of nanoparticles. **a** Side view of the internalization pathway for PEGylated nanoparticles with grafting density 1.6 chains per nm^2^. **b** Top view of the internalization pathway for PEGylated nanoparticles with grafting density 0.6 chains per nm^2^. Reprinted with permission from ref. [59]

The shape is also an important characteristic for the nanovaccines, not only because several studies indicate that nanoparticles with different shape can regulate the differentiation of immune cells, such as macrophages, but also because the shape can influence the biodistribution and uptake of nanoparticles in vivo, which can further affect the immunogenicity of the nanovaccines. Li et al. found that the spherical nanoparticles showed the fastest endocytosis rate, followed by cubic nanoparticles, then rod- and disk-like nanoparticles, as shown in Fig. 5. Through detailed free energy analysis, the nanoparticles’ shape effects can be attributed to the membrane-bending energy change. The spherical nanoparticles exhibited the minimal membrane-bending energy change, while disk-like nanoparticles displayed the maximal membrane-bending energy change. Interestingly, star-shaped nanoparticles showed similar behavior with the spherical ones in wrapping time and high efficacy for drug delivery, which can be interesting to use as guidance for the nanovaccine shape [59].

In addition to the internalized rate, other studies have focused on the internalization amount and biodistribution. Shao et al. prepared mesoporous silica nanoparticles (M-MSNPs) with a various aspect ratio (1, 2, and 4). They found that long-rod M-MSNPs exhibited the higher internalization amount in both cancer cells and normal cells than the short-rod and the sphere-like ones, because of the difference in the endocytosis pathways. A clathrin-mediated pathway was involved in the internalization of sphere M-MSNPs, while a macropinocytosis-mediated pathway was responsible for the uptake of the long-rod M-MSNPs. However, there were no significant differences in the cytotoxicity and hemolytic rate by M-MSNPs with various shapes. As for the biodistribution, through the intravenous administration, all M-MSNPs were mainly located in the reticuloendothelial system organs, including the liver, spleen, and kidney. Specifically, long-rod M-MSPs tended to travel to the spleen compared with the sphere-like M-MSNPs that were easily trapped in the liver. In addition, the rod-shaped M-MSNPs preferentially accumulated in tumor sites than sphere-like M-MSNPs [60]. The abovementioned results indicate that nanoparticle shapes interfere not only with the internalization rate and amount but also with the biodistribution, which means that the design of nanovaccines should systematically consider these interactions.

As another important feature for the nanoparticles, surface coating influences not only the hydrophilicity of nanoparticles, but also the circulation time and uptake efficacy by the phagocytic cells. Therefore, as for the nanoparticles developed as nanovaccines, the surface coating should be carefully considered. Several studies have focused on the interaction between various surface coatings, such as poly(ethylene oxide), poly(sarcosine), and hyaluronic acid, and the immune system. Many of these studies found that the PEGylation of nanoparticles, such as liposomes, and micelles can induce the generation of anti-PEG antibody by repeated injection in the animals [61]. In addition, several PEGylated products also display a decreasing therapeutic efficacy and other adverse effects after repeated administration in the clinics. Takuya et al. prepared lipid nanoparticles with different PEG lengths to investigate the relationship between PEG shedding rate and anti-PEG antibody production [62]. As reported by the Wilson et al., the length of the PEG lipophilic tail is strongly correlated with the strength of the anchor that is formed between the PEG and the lipid nanoparticle membrane, which means that the shorter the PEG acyl chains are, the faster the PEG shedding will be [63]. As a result, Takuya et al. found that the lipid nanoparticles with short acyl chain (fast-shedding) induced less anti-PEG antibody production compared with the long acyl chains (slow-shedding). In addition, the slow-shedding PEG lipid nanoparticle mainly accumulated to the Kupffer cells (resident liver macrophages) rather than the hepatocytes [57].

In addition to PEGylation, researchers have developed poly(sarcosine) and hyaluronic acid as surface coatings of nanoparticles to increase their hydrophilicity and investigated the interaction between these polymers and immunogenicity. Cheol et al. found that the nanoparticles with a long hydrophilic chain of pol (sarcosine) exhibited main accumulation on the B1a cells and the production of the class-switched antibody immunoglobulin G 3 (IgG3). In addition, the antigenicity of poly(sarcosine) and nanoparticle properties influenced the generation of IgG3 and immunoglobulin M (IgM) by different methods [64]. This result was beneficial for immunotherapy applications via antibody-dependent cell-mediated cytotoxicity. In contrast, with the antigenicity of poly(sarcosine), Abdulaziz et al. prepared chitosan nanoparticles (CS NPs), hyaluronic acid–coated nanoparticles (HA-CS NPs), and alginate-coated nanoparticles (Alg-CS NPs) [65]. They found that HA modification significantly reduced the protein adsorption on the surface of the nanoparticles. A following gene ontology analysis further confirmed that HA-CS NPs were the less immunogenic one compared with the CS NPs and Alg-CS NPs. Interestingly, two unique anti-inflammatory proteins (inter-alpha-trypsin inhibitor heavy chain and alpha-1-acid glycoprotein) were found in the protein coronas of the HA-CS NPs, but not in the Alg-CS NPs and CS NPs. In addition, a pro-inflammatory protein (Clusterin) was not found on the protein coronas of HA-CS NPs, but in the CS and Alg-CS nanoparticles.

Different nanoparticle surface coatings can lead to a difference in the composition of the protein corona, which can further contribute to the immunogenicity of nanoparticles, such as the pro-inflammatory surface coating or the anti-inflammatory coating. Therefore, when these nanoparticles are developed in nanovaccines systems, the surface coating and potential protein corona components should be carefully investigated.

### Application of nanovaccines

Inspired by the unique properties of nanovaccines, such as co-delivery of antigen and adjuvant in the same carrier, controlled release of adjuvant, activated targeting ability, and passive targeting to the lymph nodes with nanoparticles diameter at 20–50 nm, researchers have developed nanoparticles as nanovaccines for various applications, such as cancer immunotherapy and infectious disease.

When nanovaccines are applied for cancer immunotherapies, there are two main approaches in fighting against cancer cells: cell-mediated and humoral immunity [66, 67]. As for the cell-mediated immunity, the sequence of events starting with the activation of APCs, antigen process, and priming, proliferation and differentiation of T cells is the base for the effective immune function. Gao et al. prepared whole tumor cell lysates (from B16 melanoma cells) loaded nanovaccines modified with the mannose moieties (for targeting the dendritic cells). More than twofold increase in antigen uptake and maturation of bone marrow–derived dendritic cells (BMDCs) were observed in the BMDCs co-cultured with nanovaccines compared with the control group (BMDCs without any treatment). Furthermore, the nanovaccines also exhibited potent anti-tumor capability in vivo: ca. 35% of target melanoma cells were lysed by the effector T cells in the mice immunized with the nanovaccines compared with only ca. 12% in the control group [68].

In addition to the whole tumor cell lysates, tumor-related peptides are one of the most important resources for the antigens. Zeng et al. encapsulated the melanoma antigen peptide tyrosinase–related protein 2 (Trp2) as an antigen and toll-like receptor-9 (TLR-9) agonist CpG oligodeoxynucleotides (CpG ODN) as an adjuvant into a nanovaccine platform. After formulation optimization, their nanovaccines can target proximal lymph nodes and the cargo can be effectively internalized into the dendritic cells. Additionally, the nanovaccines significantly expand (more than twice compared with the free Trp2 and CpG ODN) the antigen-specific cytotoxic T lymphocytes (CTLs) and display anti-tumor efficacy in a lung metastatic melanoma model (C57BL/6 mice) [69].

Cell membrane–based biomimicking strategies provide more options to the construction of nanovaccines, since the cancer cell membranes contain plenty of tumor-specific protein that can be used as antigens in the composition of nanovaccines. In addition, some proteins on the surface of APCs cell membranes, such as costimulatory molecules, can also participate in the immune response. Specifically, our group recombined cancer cell membranes (4T1 cells) with monophosphoryl lipid A and a commercial lipid to formulate the nanovaccine liposomes (vacosomes) [70]. The vacosome-enhanced BMDC maturation (∼75% for the vacosomes and ∼13% for the untreated BMDCs) and anti-tumor ability (∼20% cell viability of 4T1 treated with the vacosomes) in vitro. Besides developing vacosomes, we also developed cancer cell membrane (MDA-MB-231 cells) coating thermally oxidized porous silicon (core structure) coated with acetalated dextran (shell structure) nanovaccines [71]. After stimulation with our nanovaccines, both the immortal cell lines and peripheral blood monocytes (PBMCs) expressed the co‐stimulatory signals (CD80 and CD86). Moreover, the nanovaccines enhanced the secretion of interferon (IFN)‐γ in PBMC and did not induce the secretion of IL‐4, which further promoted the polarization of the newly primed T cells toward a Th1 cell–mediated response. Additionally, we also observed inhibited melanoma tumor progression on mice immunized twice with the complete nanovaccine formulation in our further investigations [72]. Moreover, some researchers also attempted to fuse living cell and get the hybrid cell membranes. For example, Ma et al. fused the BMDCs (activated by lipopolysaccharides, LPS) with cancer cells (MC38 cells) [73]. In this way, the whole tumor-associated antigens and costimulatory molecules, such as CD80 and CD86, and major histocompatibility complex (MHC) II can present in the same fused cell. The authors isolated and purified the fused cell membrane and coated PLGA nanoparticles with these cell membranes (named as DMNPs in this article). As a result, they found that the fused cell membrane enhanced the accumulation of DMNPs in the spleen and lymph nodes and further elicited the T cell response. Finally, the DMNPs also exhibited potential efficacy in cancer prevention and in inhibiting cancer regression.

Besides the fusion of living cells to get the hybrid cell membranes, Yao et al. attempted to engineer the cancer cell to express a co-stimulatory marker B16-CD80/Ovalbumin (OVA) cell by transduction, and then isolate the engineering cell membranes [74]. Furthermore, they prepared the PLGA nanoparticles coated with engineering cell membranes as artificial antigen-presenting cells (aAPCs). As a result, tumor antigen-specific immune responses and priming of T cells were observed in both prophylactic and therapeutic models (C57BL/6 mice challenged with B16-OVA cells).

Considering the safety and simple production, genetic nanovaccines, such as messenger RNA (mRNA) and deoxyribonucleic acid (DNA) nanovaccines, have been widely investigated in the treatment of cancer. For example, Rein et al. prepared a nanovaccine system containing nucleoside-modified antigen-encoding mRNA (encoding tumor antigens), glycolipid antigen, and α-galactosylceramide (activation of invariant natural killer T cells, iNKT cells). A broad set of antitumor effector cells was promoted, including CTLs, iNKT cells, and NK cells, while reducing local immune suppression at the tumor site. Additionally, by combination with programmed cell death protein 1/programmed death-ligand 1 (PD-1/PD-L1) checkpoint inhibition, the nanovaccines can prevent the induction of iNKT anergy and overcome adaptive resistance at the tumor site [75]. Liu et al. generated a DNA nanodevice vaccine by precisely assembling two types of molecular adjuvants and an antigen peptide within the inner cavity of a tubular DNA nanostructure [76]. The nanovaccines opened the lysosomes in APCs exposing adjuvants and antigens to activate a strong immune response. A potent antigen-specific long-term T-cell response was observed in the B16-OVA and B16F10 tumor murine models after treatment with the nanovaccines.

**Fig. 6:**
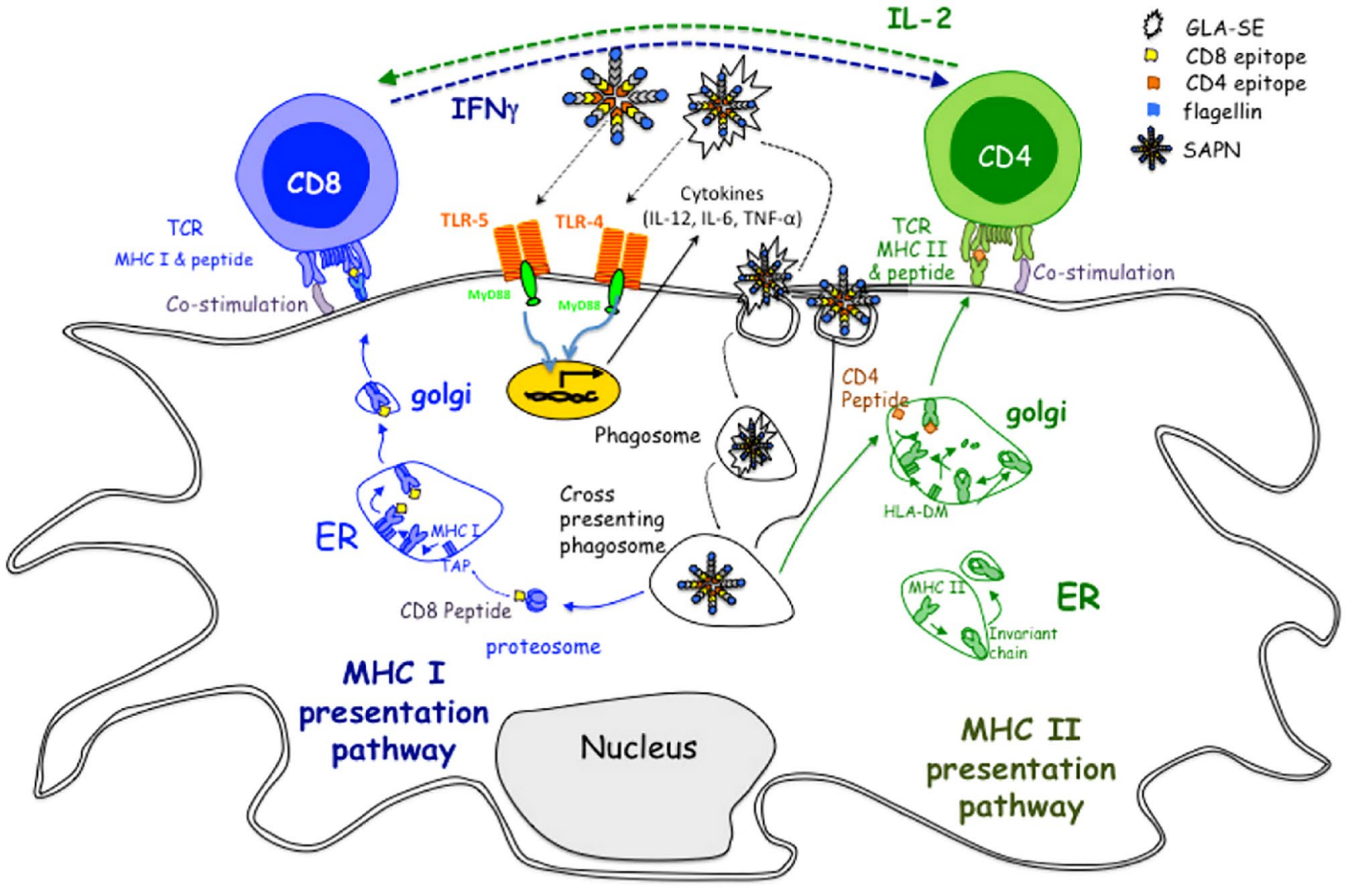
Mode of action of SAPN NPs. SAPN adjuvanted with glucopyranosyl lipid adjuvant–stable emulsion (GLA-SE) has peptides that are presented by MHC molecules on the follicular dendritic cells to T lymphocytes. Reprinted with permission from Ref. [77]

As a result of the flexible compositions, nanovaccines also exhibit potential capability in controlling infectious diseases. For example, Kamal et al. prepared a self-assembling protein nanoparticle (SAPN)-containing five CD8^+^ human leukocyte antigen (HLA)-A03-11 supertype-restricted epitopes from antigens expressed during *Toxoplasma gondii*’s lifecycle, the universal CD4^+^ T cell epitope Pan DR epitope (PADRE), and flagellin as a scaffold and TLR5 agonist, as shown in Fig. 6 [77]. After immunization with the protein nanovaccines, HLA-A*1101 transgenic mice showed effective IFN-γ responses and activated CD8^+^ T cells in fighting against *Toxoplasma gondii.*

Currently, there is still no Food and Drug Administration (FDA)-approved vaccines for the *Yersinia pestis.* Danielle et al. developed polyanhydride nanoparticle-based vaccines recombining cyclic dinucleotides and F1-V that can induce protection against pneumonic plague [78]. All the mice immunized with nanovaccines were protected from *Yersinia pestis* lethal challenge within 14 days post-immunization. In addition, after a single dose of nanovaccines, 75% of mice were still protected from a challenge even after 182 days of immunization with high levels of antigen-specific serum IgG, which demonstrated the rapid and long-lived protective immunity caused by the nanovaccine immunization.

The seasonal flu epidemics still annually cause severe illness and death around the world. Although the seasonal flu vaccine is updated annually according to the epidemic prediction and influenza surveillance data, if it mismatched with the circulating strains, the vaccines will be ineffective. Ding et al. prepared universal vaccines based on the highly conserved ectodomain of influenza matrix protein 2 (M2e) that were further inserted into capsid protein of porcine circovirus type 2 (PCV2) [79]. This nanovaccine induced high levels of M2e‐ and PCV2‐specific immune responses and protected mice from a lethal challenge of swine, human, and influenza A virus.

In conclusion, biomimetic nanovaccines are more efficient vaccine formulations because of their unusual transport kinetics, antigen profiles, immunostimulatory properties, and targeting skills.

## The future of vaccine formulations

Historically, vaccine formulation assumes that they practice equal immunogenicity and offer protection exclusively against their target pathogen regardless of the target population. However, vaccinations can have off-target effects, and the immunogenicity of the vaccine can differ significantly with demographic factors, such as age and sex. For example, epidemiological studies indicate that the value of Bacille Calmette-Guérin (BCG) vaccination can differ according to the formulation of BCG and the age of administration to optimize both unique and heterologous beneficial effects with optimum timing in early life. Overall, BCG is a precise vaccinology paradigm that will help set a standard for next-generation vaccines [80].

Recent developments in biomaterials present new possibilities for enhancing the efficacy of next-generation vaccines. Most current vaccine technologies are poorly immunogenic, have only intermittent protection, or generate chances of regaining pathogenicity. Strong collaborative efforts among researchers in different fields would lead to new biomaterials with improved properties. New physical and chemical structures would play critical roles in vaccine safety, cellular trafficking, and overall immune response [81].

The COVID-19 pandemic caused by the SARS-CoV-2 virus is a clear warning that new infectious diseases with pandemic potential can inflict high human and economic losses. In response to the crisis, regulatory agencies have made unprecedented strides to help get safe and reliable vaccinations to the market sooner [82]. Leaders must invest in evidence-based vaccine delivery strategies that generate demand, allocate, and distribute vaccines. Verifying coverage is essential to have a widely immunized population [83]. Researchers have previously studied the difficulties of supplying communities with life-saving equipment or drugs. The phenomenon of “implementation bottlenecks” is a leading cause of the inability to convert recognized measures into robust service delivery [84]. It is fundamental to establish a rigorous communication strategy to encourage vaccine uptake, particularly in communities that we would imagine will be reluctant or reject vaccination [85]. Industries are essential collaborators in all attempts to plan for and best adapt to epidemics, pandemics, and emerging infectious diseases. Therefore, the global community has an opportunity to build on this momentum to design a sustainable model for vaccines [82].

## A new vaccine delivery platform: microneedles

### Microneedle technology

In this review, we assessed vaccines as a vital component of pandemic preparedness. Insufficient manufacturing capability can hinder its production and delivery. Also, there is a strong need to establish technologies to reduce the antigen dose, since lower vaccine antigen doses led to better T cell responses. In vitro studies showed a close relationship between antigen dose and functional avidity of CD8 T cells. Recent data have confirmed this relationship for CD4 and CD8 T cells after vaccination in both animals and humans [86]. The exploration of such dose-saving strategies requires alternate routes for the administration of vaccines, such as intradermal. Conventional vaccine injection bypasses the skin’s immune system and introduces the antigen into the muscle or subcutaneous tissue where there is no detectable resident APC population. However, the skin is an anatomical area with greater immunogenicity capacity due to the presence of many of epidermal Langerhans cells and dermal dendritic cells [87]. A Phase I trial was performed by Combadière et al. to prove intradermal superiority relatively to intramuscular. The transcutaneous delivery of the inactivated influenza vaccine resulted in a more effective induction of influenza-specific CD8 + T cell responses relative to that administered through the IM route [88]. Despite the advantages and substantial research efforts, transdermal vaccine delivery has yet to reach its full potential as an alternative to hypodermic injections [89].

**Fig. 7:**
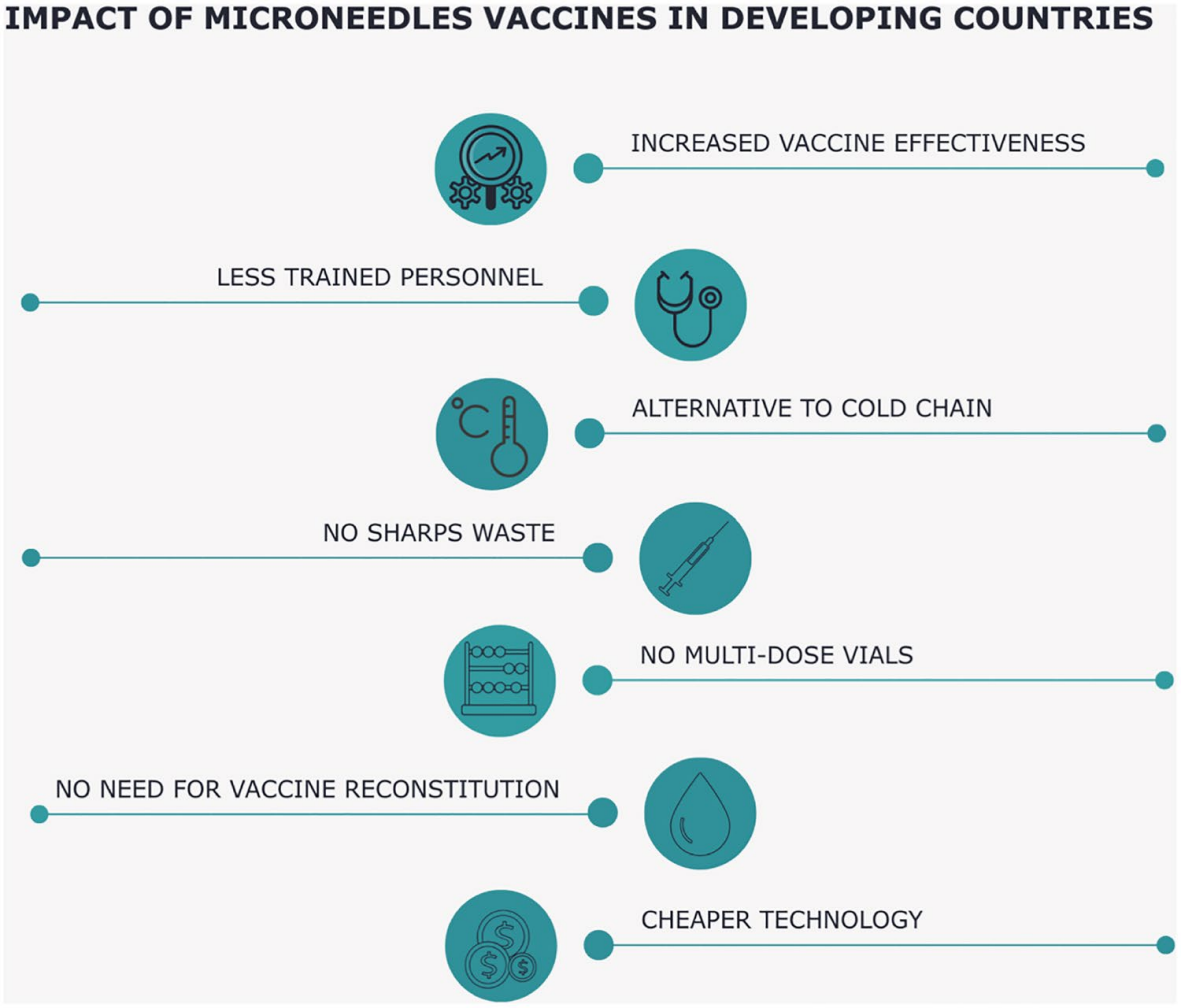
Possible benefits of MN vaccine implementation in developing countries

An obvious obstacle to injections is needle phobia. Survey reports that ~25% of parents and more than 60% of children have reported fear of needles, a significant barrier to vaccination [90]. Since MNs are short and narrow enough to prevent dermal nerve stimulation, there is no pain associated with administering vaccines through this route [91]. MN-based patches aim to resolve the need to deliver drugs with ease of oral administration and effectiveness equal to injection [92]. MNs measure hundreds of microns and are administered in the skin, which is 1–2 mm thick at specific administration sites, overcoming the outermost skin barrier layer, the stratum corneum, and providing transient pathways for minimally invasive transcutaneous delivery. Specifically, an array of MNs is attached to a backing, permitting bandage-like application. Therefore, administering of vaccines through the skin using MNs would present a cost-effective, quick, and secure solution without trained personnel (Fig. 7) [93].

Microneedle (MN) vaccination provides a superior immunological response at the same dose. Several studies have found higher antibody production and better cellular response using MNs compared with hypodermic injections [94–96]. Using a very dense array of MNs may enhance the immunological response. More significant damage to cells in the epidermis would increase the immunogenic signal, leading to further savings in vaccine dosage. By reducing antigen exposure to protein-damaging stresses during traditional microparticle fabrication/antigen encapsulation, antigen stability can be favorably preserved [97]. Existing MNs address skin-resident dendritic cells directly; antigens distributed by MNs are taken-up by skin dendritic cells and transported for antigen presentation to the cutaneous draining lymph nodes [93, 98]. As recent studies have shown, the latter is a problem, since these dendritic cells are relatively inefficient in antigen transport, as less than 1% of the injected antigen enters lymph nodes [99]. An alternative approach can be to deliver antigens where they can be drained to lymph nodes and activate lymph node–resident dendritic cells. The dermis layer of the skin is highly perfused with lymphatic capillary networks. Consequently, directly targeting vaccines to lymph nodes following intradermal delivery via MNs seems plausible [100]. In a study related to MN protein delivery, Harvey et al. conducted imaging studies using reporter dyes showing rapid lymphatic-mediated uptake [101].

MN improves vaccine compliance and can improve safety by minimizing the production of harmful medical waste, inhibiting disease transmission through needle reuse and needle-based accidents. The latest research has shown the effectiveness of MNs for stratum corneum reliable and pain-free disruption, encouraging transcutaneous delivery of a broad spectrum of vaccine components [102, 103]. It is critical, though, to perform further research into adverse reactions and side effects, to gain full understanding of the long-term consequences of polymer deposition in the skin. Although repetitive long-term vaccine delivery applications are unnecessary, there is the need to thoroughly elucidate polymer deposition effects. Polymer deposition can result in polymer accumulation in the tissue, causing local erythema or developing granuloma or accumulation in the body’s clearance organs [104].

**Fig. 8:**
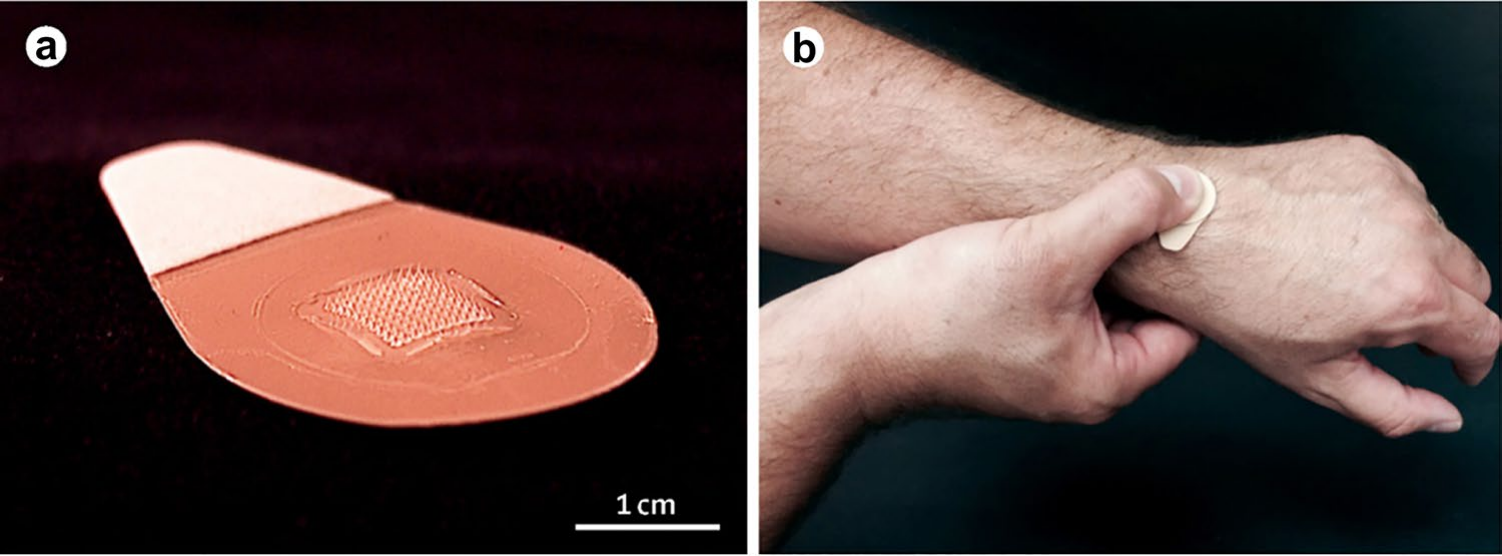
MNs for influenza vaccination. **a** MNs contain an array of 100 MNs measuring 650 μm tall that is mounted on an adhesive backing. **b** MNs are manually administered to the wrist, enabling self-administration by study participants Reproduced with permission from Ref. [107]

A potential advantage of MNs is that they would reduce the expertise needed for administration, because they are pressed to the skin by hand or using a specific applicator (Fig. 8) [92, 105]. Self-administration is not appropriate in some cases, but administration by less-trained personnel would still boost access to vaccines, especially in developing countries [92, 106].

Another issue in these countries is vaccine wastage. For example, sometimes only a portion of the doses is used before the vaccine expires. It can also happen that health care personnel decides not to vaccinate a patient, because doing so would require opening a new vaccine vial when there are not enough patients to use the entire vial [92, 108]. Avoiding these complications is possible by using disposable single-dose MNs. The “cold chain” is a further core aspect when delivering vaccines from the point of manufacturing to the point of use. The cold chain is a costly method to store and distribute vaccines at recommended temperatures to preserve vaccine potency. The estimated cost of cold chain storage is $200–300 million annually, and vaccine shortages frequently result from this infrastructure’s deficiencies [109].

Although maintaining the cold chain is expensive, it is crucial in supplying vaccines to distant clinics in hot climates served by poorly developed transport networks [38]. MNs can have improved stability without refrigeration, in part because of their solid-state nature [92]. Mistilis et al. demonstrated that a MN-based vaccine for influenza can be stable at least 6 months at 25°C and at least a few weeks at 40°C [110]. Other studies had similar results with different formulations [111, 112].

Lyophilized vaccines are much more amenable to long-term storage and more resistant to temperature extremes and humidity. Freeze-drying might therefore help in conditions where maintaining the cold chain is difficult, but the solid phase must then be followed by a reconstitution back into a ready-for-injection liquid form. It is a delicate procedure that requires specialized personnel to avoid errors that could have severe consequences on patients’ health. On the contrary, MNs do not need to be reconstituted, as they use the fluids of the skin to deliver the molecules [113]. MNs still need conditions to maintain drug activity, integrity, and sterility. Packaging plays a considerable role in providing chemical, physical, and mechanical protection [92].

All the mentioned positive attributes of MNs must be rigorously evaluated and addressed to translate the technology into clinical usage. End-user adoption of this technology must be measured, understood, and modified to incorporate users’ needs [114]. A recent literature review analyzed MN vaccination technology perception and acceptance, especially in the pediatric population. The findings revealed favorable views of the general population and health care practitioners’ technology, mentioning several benefits widely associated with this technique. Even so, there were questions of unfamiliarity with the technology and ability to ensure accurate delivery of vaccines [115].

A current issue is that majority of MN studies took place on a laboratory scale. In order to scale-up the production, new approaches to manufacturing need to be developed, and further financial investments from large pharmaceutical are needed over the coming years. Dose loading capability must also be discussed for the manufacturing considerations of MNs so that the vaccine manufacturer can integrate the technology into its processes [116]. Centrifugation is still used by most laboratories, but automatic nanodispensers or other faster methods may be a more adequate solution for industries. For example, a team developed a new kind of female mold, namely, a double-penetration female mold (DPFM). DPFM has the pinpoints covered by a waterproof breather membrane that has proven helpful in reducing the effect of gas resistance and viscosity of the solution. For the scale-up fabrication of dissolving MNs, a positive-pressure microperfusion technique (PPPT) based on DPFM was proposed [117]. Finally, establishing uniform approval requirements and standards for good manufacturing practice, permitting MN characterization and eventual commercialization, is essential [118].

MNs have many excellent features, such as the ability to cross the stratum corneum without pain, minimal invasiveness, and the ability to skip the first-pass metabolism. Such benefits make MNs outstanding candidates for immunological biomolecule delivery [119].

### MN-based formulation

Solid MNs generate micropores in the skin, and after removal of the MNs, a patch is applied, allowing the drug in the patch to diffuse. Disconnected treatments can be an incommodious process, and intradermal injection of the vaccine was more effective than solid MNs [113]. For these reasons, our review will not focus on solid MNs, but on the most used MNs for vaccines: coated and dissolving. Dissolving MNs are the most advanced and complex, as they can encapsulate vaccines within their matrix [120], while the MN shaft and tip sizes constrain the delivery of vaccines via coated MNs [121]. Both types use biocompatible polymers and exploit the fluids present in the skin for dissolution to deliver the payload. In coated MNs, only the coating dissolves, while in dissolving MNs, the MNs dissolve entirely, leaving the backing intact and with no sharp residual [113]. However, the small size of MNs tips usually restricts dosing to less than 10 mg and ideally less than 1 mg. The bright side is that vaccine doses are typically less than 0.1 mg [92].

The most common technique for manufacturing polymer MNs includes using an inverse mold, usually made of polydimethylsiloxane. The protocol consists of casting the polymer solution and drying it within the mold. The last stage is to peel MNs off from the mold after drying [122]. The choice of materials for the MNs must satisfy multiple parameters, such as the need to be sufficiently strong to puncture the skin and be acceptable for manufacturing processes using current good manufacturing practice. In other examples, MNs are made of swellable hydrogels that release the encapsulated drug upon gel hydration [92]. Arya et al. used dissolving MNs to administer rabies DNA vaccine to dogs. They administered the patches manually, and the MNs dissolved in the skin in about 15 min [123]. Another study used a coating of PLGA-PLL/γPGA nanoparticles to deliver an Ebola DNA (EboDNA) vaccine. The resulting coated MNs showed a higher EboDNA loading and greater mechanical strength than the ones with a naked EboDNA coating [124].

### MN-based applications

In the previous section, we mentioned examples of DNA vaccines, but the compatibility of MNs goes way beyond this; it has also been proven with subunit, virus-like particles, live-attenuated, and inactivated vaccines. For example, MN-soluble glycoprotein subunit vaccines are another possible approach to achieve protection against the lethal Ebola virus [125]. Zhu et al. encapsulated enterovirus 71 (EV71) recombinant virus-like particles in MNs, to investigate the immune responses against EV71 infection [126]. Fabricated MNs dissolved and released payload rapidly within 2 min of application, and the punctured skin displayed just mild erythema that healed rapidly 24 h after treatment with MNs. More specifically, immunization tests in mice have shown that tenfold lower antigen dose–loaded EV71 MNs produced intense immune responses and defensive efficacy comparable with traditional IM injection.

**Fig. 9:**
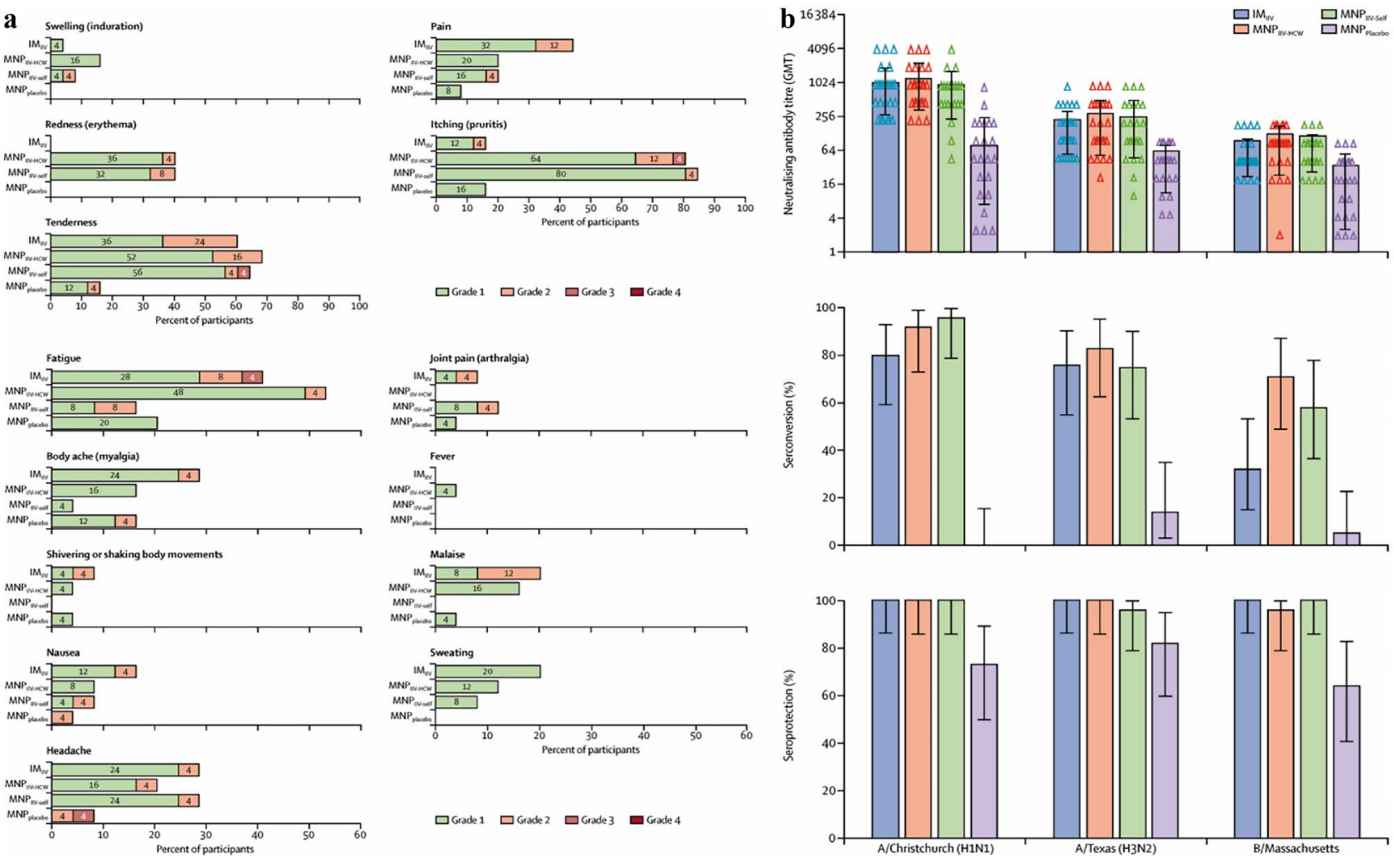
Solicited reports of adverse events 7 days after vaccination and serological response to study drug administration. **a** Local and systemic adverse events associated with vaccination are shown in different groups. IIV inactivated influenza vaccine, 7 days after vaccination, MNP microneedle patch, HCW health-care worker, IM intramuscular. **b** Hemagglutination inhibition GMTs (log 2), seroconversion, and seroprotection against A/Christchurch/16/2010 (NIB-74 [H1N1]), A/Texas/50/2012 (NYMC X-223 [H3N2]), and B/Massachusetts/2/2012 (NYMC BX-51[B]) strains for MNP_IIV-HCW_, MNP_IIV-self_, MNP_placebo_, and IM_IIV_ 28 days after vaccination. Bars show 95% CI. GMT geometric mean titers, IIV inactivated influenza vaccine, MNP microneedle patches, HCW health care worker, IM intramuscular Reproduced with permission from ref. [107]

Live-attenuated viruses are unstable above 8°C, so a cold chain is necessary, making them pricey [127]. This represents an appealing case for the application of MNs. One study demonstrated that intradermal administration of live-attenuated herpes zoster vaccine resulted in increased varicella-zoster virus-specific antibody production at the same dosage of subcutaneous injection [128]. The same study hypothesized that mild inflammation following intradermal vaccination might increase the adaptive immune response’s efficiency. However, the research is based on a limited sample size and has found no significant cell-mediated immunity variations [129]. Erdos et al. instead found a more robust cellular immune response, using polyinosinic acid, polycytidylic acid as an adjuvant in combination with an adenovirus-encoded antigen. Lastly, a clinical trial with inactivated influenza vaccine delivered by MNs was conducted in humans [107]. MNs were well tolerated, well accepted and result in robust immunological responses whether administered by healthcare workers or by the participants themselves (Fig. 9). These findings showed that MN vaccination could be an exciting new method to increase the present-day availability of vaccines and reduce immunization costs.

The spectrum of pathogen vaccines tested with MNs is also quite broad and includes bacteria, protozoa, and viruses. A limited number of studies have investigated MN vaccination against bacteria. Live-attenuated Bacille Calmette-Guerin (BCG) bacillus is the only tuberculosis-preventive vaccine approved worldwide to date. This specific vaccine must be delivered intradermally, causing severe skin inflammation and, sometimes, permanent scars. Chen et al. developed MNs with an internal “cave” to accommodate and release the powder BCG vaccine into the intradermal space [130, 131]. The approach managed to avoid any adverse reaction, achieving an immune response comparable to IM vaccination. Based on a previous publication, which provided an essential proof-of-concept regarding the delivery of non-toxoid bacterial antigens [132], Pastor et al. conducted a study for MN vaccination against Shigellosis [131]. To further prove the MN-based technology’s versatility, a group developed a dissolving MNs device called MicroHyala and tested its efficacy against tetanus and diphtheria, malaria, and influenza [133]. The findings indicated effective immune responses against all infectious diseases. Researchers have also investigated the use of MNs to deliver alternative and model vaccine agents, in addition to the delivery of vaccines currently in use. A study proved that delivering DNA vaccines might provide a promising approach to cancer immunotherapy. The group loaded a synergistic nanopolyplex cocktail on dissolving MNs, succeeding in highly efficient use of this non-invasive and straightforward delivery system, overcoming the weaknesses of traditional injections [134].

Moreover, the use of MNs has been investigated by researchers working on a possible vaccine against the current coronavirus causing the COVID-19 pandemic. It is unlikely that the industry will decide to bet on a new technology immediately after a new vaccine approval, but it cannot be ruled out that MNs may be used for future generations of COVID-19 vaccines. Using the existing experience with MERS-CoV vaccines, Kim et al. performed the first study on a possible MN COVID-19 vaccine with SARS-CoV-2 S1 subunit vaccines [135]. MN-based patches were added to the skin of mice, collecting serum at various times. By week 2, the group observed elevated levels of virus-specific IgG. The gamma-irradiation sterilization of the patches did not affect immunogenicity. MN delivery of coronaviruses-S1 subunit vaccines is a promising immunization strategy against coronavirus infection.

Overall, it is indisputable that the skin, the largest organ in the human body, has become an attractive vaccine delivery site. The large number of publications and new groups studying MNs is further evidence of this technology’s enormous potential for vaccine delivery. In particular, MNs seem to offer possible solutions to face vaccine formulations’ biggest problem that is maintaining vaccine component stability, both during manufacture and during storage. There is no doubt that further clinical vaccination studies in human volunteers are needed to demonstrate safety and to prove the efficacy of this vaccine approach further. However, there is great optimism in the scientific community regarding the significant impact that vaccine-loaded polymeric MNs may have on global health. Both scientific and technological efforts will enable quicker responses to emerging pandemics [136].

## Conclusions

With the advent of new health conditions that cannot be addressed by traditional methods, it has become necessary to explore new areas regarding vaccination. The advantages offered by nanoparticles’ proprieties have been widely exploited in the formulation of new vaccines. Several clinical studies have proven their efficiency in enhancing both cellular and humoral immune responses. The possibility of formulating vaccines in the dry state remains one of the most coveted objectives, because it would allow circumventing the need for the cold chain. A dry formulation and the possibilities of reducing the number of qualified personnel are two of the primary reasons for the scientific community’s interest in microneedle-based vaccines. Although clinical trials have begun, there is still a need to expand the number and variety of patients, and to develop a technology that has a broad spectrum of applicability. Therefore, it is undeniable that future vaccination depends on the success of research in developing long-term preservable vaccines, easily administrable, and guarantees a strong and lasting immune response.

## Data Availability

Data sharing is not applicable to this article, as no datasets were generated or analyzed.
